# Improved Detection of Bifidobacteria with Optimised 16S rRNA-Gene Based Pyrosequencing

**DOI:** 10.1371/journal.pone.0032543

**Published:** 2012-03-28

**Authors:** Kathleen Sim, Michael J. Cox, Harm Wopereis, Rocio Martin, Jan Knol, Ming-Shi Li, William O. C. M. Cookson, Miriam F. Moffatt, J. Simon Kroll

**Affiliations:** 1 Imperial College London, St. Mary's Campus, London, United Kingdom; 2 Imperial College London, National Heart and Lung Institute, South Kensington Campus, London, United Kingdom; 3 Danone Research, Centre for Specialised Nutrition, Wageningen, The Netherlands; University of Hyderabad, India

## Abstract

The 16S rRNA gene is conserved across all bacteria and as such is routinely targeted in PCR surveys of bacterial diversity. PCR primer design aims to amplify as many different 16S rRNA gene sequences from as wide a range of organisms as possible, though there are no suitable 100% conserved regions of the gene, leading to bias. In the gastrointestinal tract, bifidobacteria are a key genus, but are often under-represented in 16S rRNA surveys of diversity. We have designed modified, ‘bifidobacteria-optimised’ universal primers, which we have demonstrated detection of bifidobacterial sequence present in DNA mixtures at 2% abundance, the lowest proportion tested. Optimisation did not compromise the detection of other organisms in infant faecal samples. Separate validation using fluorescence *in situ* hybridisation (FISH) shows that the proportions of bifidobacteria detected in faecal samples were in agreement with those obtained using 16S rRNA based pyrosequencing. For future studies looking at faecal microbiota, careful selection of primers will be key in order to ensure effective detection of bifidobacteria.

## Introduction

With the advent of next-generation sequencing, semi quantitative, in-depth characterisation of microbial communities that has never been practically possible is now becoming increasingly accessible to researchers. In samples from the gastrointestinal (GI) tract, use of universal primers for amplification of the bacterial 16S rRNA gene followed by pyrosequencing is beginning to reveal the role of the GI microbiome in diverse diseases such as obesity [1], atopic disease [2], [3], colonic cancer [4] and necrotizing enterocolitis [5]. Two of the key questions surrounding the role of the GI microbiota in health are how the microbiota is involved in immunomodulation [6], [7], and how imbalance may lead to disease states. Organisms such as the bifidobacteria, which rapidly colonise the gastrointestinal microbiota in the first year of life are thought to be central in the establishment and maintenance of a ‘healthy microbiota’ in later life.

Universal PCR primers allow amplification, and therefore detection of all the bacteria in a mixed population. A number of primer sets amplifying different regions of the 16S rRNA gene exist and are in common use [8], [9]. A truly universal primer pair that binds to the 16S rRNA of all eubacteria is impossible to design since the longest number of consecutive nucleotides in the gene that are 100% conserved is 11 (*Escherichia coli* 16S rDNA positions 788 to 798), and in general, the number of sequential absolutely conserved nucleotides in other regions of the gene is four [10]. The decreased amplification efficiency due to differential annealing of universal primers when a heterogeneous template is used leads to bias against the detection of certain taxa [11]. For example, even well designed primers matching over 95% of sequences in the Ribosomal Database Project (RDP) [12] from the dominant bacterial phyla present in the gut, may miss specific taxa; primer 967F [13] will detect less than five percent of Bacteroidetes whilst primer 1492R [14] detects only 61% of Actinobacteria and 54% of Proteobacteria [15]. Mismatches towards the 3′ end are likely to lead to greater amplification inefficiency than that at the 5′ end [16]. Pragmatic approaches to primer use are often taken, accepting that not all bacteria will be fully represented, but that between sample comparisons making use of the same primer pair are valid and that particular organisms of interest are successfully amplified.

In order to address this issue, different approaches may be adopted to ensure that detection of the specific taxa of interest to the study are maximised. The universal primer set used can be optimised by either introducing a degenerate base pair at the positions of mismatch. Alternatively, taxa-specific primers can be added to the primer pool. Frank, et al. [16] used a primer pool consisting of seven different primer sequences (fourfold-degenerate primers and three primers specific for amplifying *Bifidobacteriaceae, Borrelia and Chlamydiales*) and were able to dramatically increase the detection of genera which were previously missed from clinical samples. Increasing the number of degenerate bases in the primer set may however introduce a bias in the template to product ratios when a heterogenous template is used since templates with a greater GC content at the primer site will be preferentially amplified [17]. Furthermore, inclusion of a large number of degenerate bases equates to dilution of the primer pool, and the number of templates which exactly match each primer sequence is reduced, resulting in a potential decrease in the overall annealing efficiency [16]. Using an inosine residue at the mismatched positions is an alternative approach [10], but as it forms a stable bond with all four nucleotides, this may lead to erroneous PCR products [16].

### Bifidobacteria

Bifidobacteria are considered to be a major component of the GI microbiota in healthy breast-fed infants [18], [19]. This is mainly driven by a high level of complex oligosaccharides (10–12 g/L) available as a natural prebiotic in human milk [20]. Their use as a probiotic, or their stimulation by adding prebiotics (synbiotics) has become increasingly widespread. Specific prebiotics or synbiotics added to infant milk formula have been shown to induce a microbiota similar to that of breastfed infants with associated physiological changes (metabolic end products and pH) compared to standard formula [21], [22]. These changes are considered as an important mechanism for the inhibition of pathogens in the gut [23]. Used as a prophylactic infant feed supplement bifidobacteria have been found to be effective at reducing both the severity as well as the risk of developing rotavirus diarrhoea. Their use also appears to reduce the risk of antibiotic-associated diarrhoea [24]. Moreover, bifidobacteria may be beneficial in the treatment of atopic disease [25] and a synbiotic infant formula has been found to prevent asthma-like symptoms in infants with atopic dermatitis [26].

Bifidobacteria were found to constitute only a minor component of the faecal microbiota in healthy, full term infants [27]. The authors acknowledge that this was surprising and speculated that this result might arise through the 8F universal primer having a three base pair mismatch against *Bifidobacterium longum*, and that the genus in general does not have 100% sequence identity to the 8F primer sequence. In our study, we have therefore sought to assess the impact of using a standard ‘universal’ primer set with one exactly matched to the target region of bifidobacteria, in detecting this genus.

We designed a ‘bifidobacteria-optimised’ universal primer set by modification of a well established primer set 357F/926R, originally designed by the Muyzer group [28], [29] for denaturing gradient gel electrophoresis. Primer set 357F/926R is one of two primer pairs recommended by the NIH Human Microbiome Project protocols [30], [31] for 16S rRNA amplicon pyrosequencing. We demonstrate that our ‘bifidobacteria-optimised’ primer set increased the bifidobacteria detection rate in both pure DNA mixtures as well as faecal samples, without compromising the detection of other genera. In addition, we have independently confirmed the relative abundance of bifidobacteria detected using fluorescence *in situ* hybridisation (FISH).

## Results

### Pyrosequencing

Pyrosequencing of the standard mixes and the faecal samples was carried out in a single multiplexed run on the GS Junior platform and resulted in 85 126 reads. After denoising and chimera-removal 60 794 high quality reads remained and these were assigned to samples using the barcode sequences, 37 977 reads for faecal samples, 22 817 for the standard DNA mixtures.

### DNA mixtures

Standard universal primers detected *Streptococcus pneumoniae* and *Moraxella catarrhalis* sequences in correct relative proportions in the DNA mixtures. The primers however, consistently failed to correctly quantify the bifidobacterial sequences present. The standard universal primers failed to amplify bifidobacterial DNA to a level above 1% in four out of the five mixtures, and the maximum proportion of bifidobacteria that was detected was 1.6%, even when the bifidobacterial DNA constituted 90% of the mixture.

**Figure 1:**
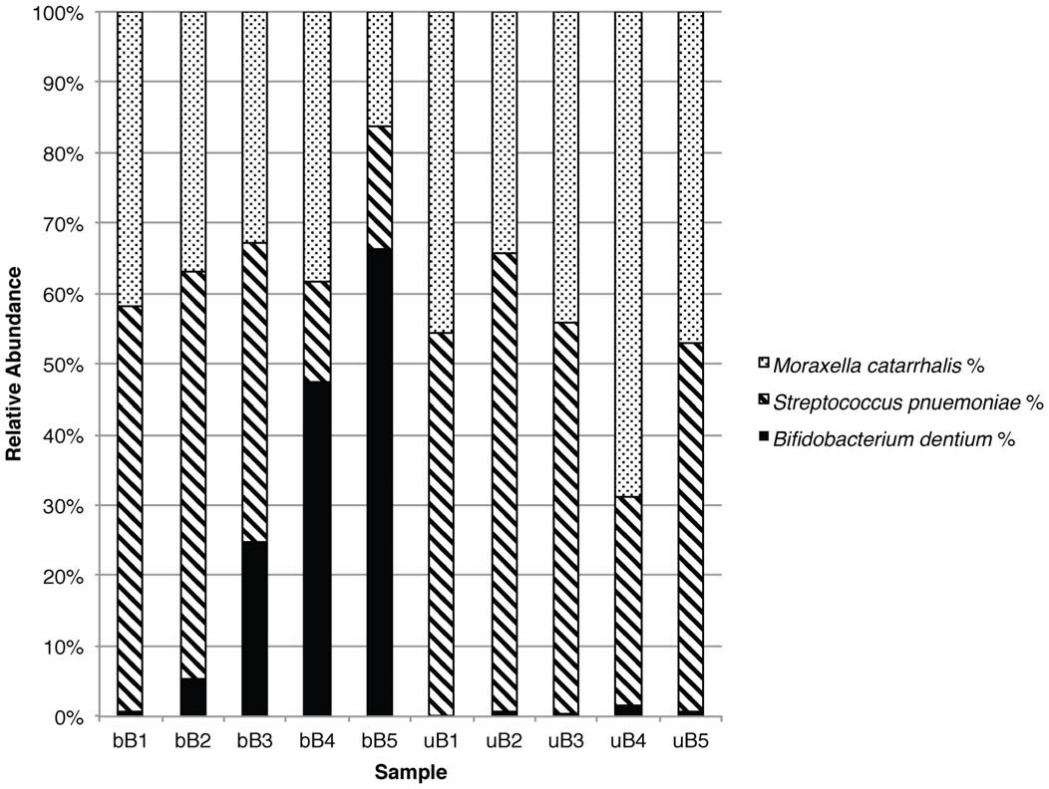
Proportions of 454 sequencing reads obtained using both primer sets. Increased detection rate of *Bifidobacterium dentium* demonstrated using the ‘bifidobacteria-optimised’ universal primers (b) compared to regular universal primers (u).

This was in contrast to the relative proportions of species-specific reads obtained with ‘bifidobacteria-optimised’ universal primers, which correlated far better with the original proportions of the species' DNA in the mixture (R^2^ = 0.955) (**Table 1** **,** **Figure 1**). With the ‘bifidobacteria-optimised’ primers, bifidobacterial DNA could be detected at the lowest concentration tested (2%).

**Table 1:** Proportions of DNA in each mixture.

Sample	*Bifidobacterium* *dentium*	*Streptococcus* *pneumoniae*	*Moraxella* *catarrhalis*
1	2%	49%	49%
2	15%	50%	35%
3	50%	25%	25%
4	75%	5%	20%
5	90%	5%	5%

### Faecal samples

#### Operational taxonomic unit (OTU) analysis

**Figure 2:**
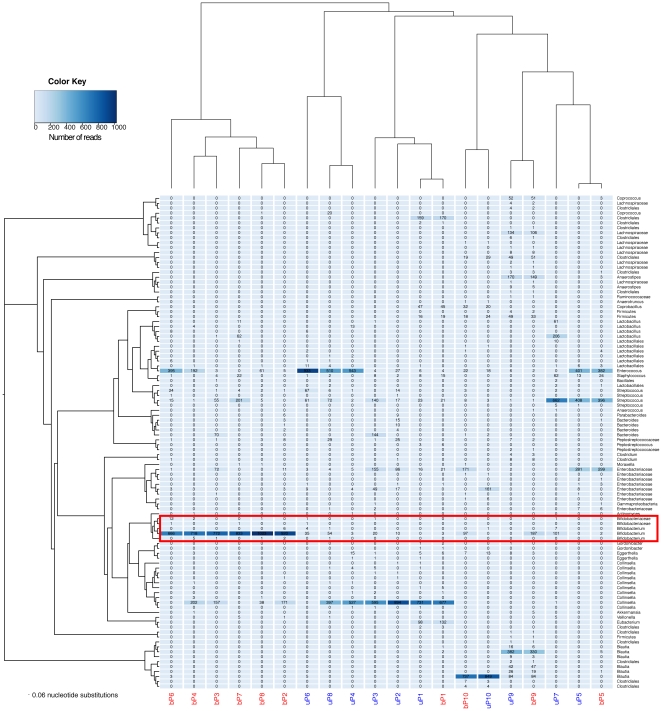
Heatmap displaying the relative abundance of OTUs per sample. Samples are grouped by hierarchical cluster analysis on the x-axis and by neighbour-joining phylogenetic tree with nearest neighbour interchange on the y-axis. Samples amplified with ‘bifidobacteria-optimised’ primers are in red and with the standard primers in blue. Bifidobacterial OTUs are highlighted in the red box.

The most abundant taxa at phylum level were the Firmicutes and Actinobacteria, followed by Proteobacteria and Bacteroidetes, irrespective of which primer set was used. The ten samples all comprised of different numbers of OTUs and OTU abundances (**Figure 2**), but, the most striking difference was the increased number of bifidobacterial reads present in the sample set analysed with the ‘bifidobacteria-optimised’ universal primers.

#### Fluorescence in situ hybridisation (FISH) analysis

**Table 2** shows the proportion of faecal bifidobacteria, expressed as a percentage of the total number of bacteria in faeces as enumerated by FISH and the relative read abundances by 454-sequencing.

**Table 2:** Relative proportions of faecal bifidobacteria in ten faecal samples as determined by FISH and 454-sequencing using ‘bifidobacteria-optimised’ universal primers (926Rb) or regular universal primers (926R).

Sample	926Rb	926R	FISH
P1	0.2%	0.0%	0.3%
P2	81.1%	1.0%	61.2%
P3	69.0%	1.7%	70.9%
P4	63.5%	0.4%	75.8%
P5	0.2%	0.0%	0.6%
P6	62.7%	4.4%	67.3%
P7	74.1%	10.8%	47.5%
P8	90.6%	5.3%	75.0%
P9	16.9%	0.0%	10.4%
P10	8.0%	0.1%	67.0%

Comparing data obtained with the two primer sets to the FISH using Pearson correlation shows significant correlation of FISH with the pyrosequencing using the ‘bifidobacteria-optimised’ primer set (**Table 3**). To confirm good agreement between two methods Bland-Altman agreement tests were performed [32]. The agreement between two methods is tested by comparing the differences between two methods against the average of the methods. The results from bifidobacteria-optimised pyrosequencing against the FISH method shows agreement in determining the level of bifidobacteria in the faecal samples tested (**Table 4**).

**Table 3:** Correlation matrix (Pearson) shows the Pearson correlation coefficients and p-values.

Variables	926Rb	926R	FISH
926Rb	n/a	0.593 (p = 0.071)	**0.761 (p = 0.011)**
926R	0.593 (p = 0.071)	n/a	0.297 (p = 0.404)
FISH	**0.761 (p = 0.011)**	0.297 (p = 0.404)	n/a

Values in bold are different from 0 with a significance level alpha = 0.05.

**Table 4:** *P*-values resulting from Bland-Altman agreement tests.

Variables	926Rb	926R	FISH
926Rb	n/a	**0.0026**	0.8974
926R	**0.0026**	n/a	**0.0011**
FISH	0.8974	**0.0011**	n/a

Values in bold are different from 0 with a significance level alpha = 0.05.

#### Principal Coordinate Analysis and statistics

**Figure 3:**
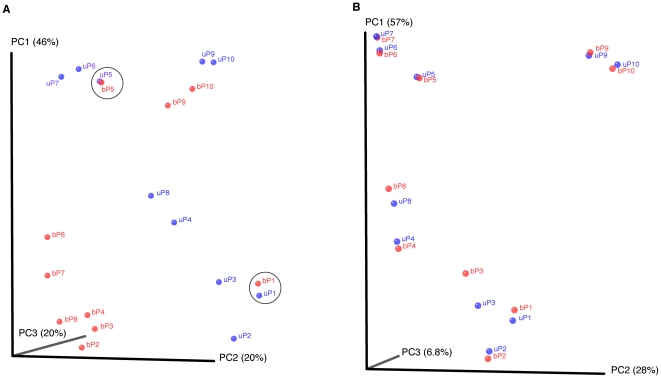
Principal Coordinate Analysis using the weighted UniFrac metric. (**A**) Sample pairs P9, P10 and in particular P1 and P5 cluster tightly together. These samples contain small or moderate numbers of bifidobacteria reads. (**B**) After removing bifidobacteria sequences from the analysis, all sample pairs cluster tightly showing that the main differences between the sets are due to the bifidobacteria sequences. U = regular universal primers (926R), B – ‘bifidobacteria-optimised’ universal primers (926Rb).

In order to ensure that detection of other organisms was not compromised or that abundance levels were not altered by using ‘bifidobacteria-optimised’ primers, principal coordinate analysis (PCoA) was performed. PCoA using the weighted UniFrac metric [33] (**Figure 3a**) (which takes into consideration both the presence/absence as well as abundance of sequences,) demonstrates clustering of samples by primer set used except for pairs P1 and P5 (circled). On OTU analysis, (**Figure 2**) these are shown to have very small or only moderate numbers of bifidobacteria present. Removing bifidobacterial sequences from the principal coordinate analysis (**Figure 3b**) resulted in tight clustering of *all* pairs of samples. This indicates that the main differences between the two principal coordinate analyses are due to the detection of bifidobacteria, and that ‘bifidobacteria-optimised’ universal primers do not compromise the quantitative detection of other organisms.

Using a paired T-Test to compare OTUs and read abundance of the two sample sets (‘bifidobacteria-optimised’ universal primers vs. regular universal primers) there was a highly significant difference between the read abundance of bifidobacteria using ‘bifidobacteria-optimised’ primers compared to regular primers (*P* = 0.039, t = 0.0026, with Bonferonni correction for multiple testing), but no significant differences between any of the other OTUs (*P*>1.4).

### Primers

Primer specificity of the 926Rb primer was compared *in silico* against that of 926R using the Ribosomal Database Project's (RDP) Probe Match tool. Only sequences longer than 1200 bp, defined as good quality by the RDP were included and 92.4% of these were hit with 0 mismatches with primer 926R compared to 94.5% with 926Rb. Although this overall increase was modest, the difference on looking specifically at the order Bifidobacteriales was very marked and highly significant: 926R hit just 0.2% of sequences compared to 97.1% with the ‘bifidobacteria-optimised’ primer.

## Discussion

Appropriate primer selection in microbiota studies using a 16S rRNA approach is essential to enable faithful representation of the organisms present in the samples. The study of Palmer, et al. [27] revealed that the overall efficiency of amplification of DNA from bifidobacterial species was eight fold lower than that from non-bifidobacterial species using the 8F/1391R primer pair. Our results show that even a one base pair mismatch not at the 3′ end of a primer can lead to a dramatic failure to amplify these organisms at all.

It is well known that Gram-positive organisms (such as bifidobacteria) can be underrepresented in microbial profiling studies due to the presence of their thick cell wall [34]. Due to concern that poor representation of bifidobacteria from faecal samples may be due to difficulties in cell lysis during DNA extraction, we first assessed target sequence recovery from pure DNA mixtures. We were able to demonstrate with the DNA mixtures that the bias observed against the detection of bifidobacteria was due to the PCR step. This was also confirmed by using FISH analysis which does not require cell lysis. From the FISH results, the bifidobacteria proportions present in the faecal samples were in agreement with those generated from our robust DNA extraction method combined with our ‘bifidobacteria-optimised’ universal primers and pyrosequencing.

Burgeoning interest in the development of the normal GI microbiota, and its impact on child and adult health, has led to increasing numbers of studies focusing on the bacterial colonisation of the gut [7]. Metchnikoff's [35] suggestion that it is “possible to adopt measures to modify the flora in our bodies and to replace the harmful microbes by useful microbes” over a hundred years ago has led to the concept of manipulating the GI microbiota to counter disease. Furthermore, the use of probiotics as a treatment or prophylaxis strategy not only for disease, but also for modulating the immune system has now become a focus of intense attention [36]. Due to the escalating use of probiotics, the World Health Organization have published specific criteria that a probiotic must fulfil [37]. One important quality of a probiotic is that it must be able to survive the GI tract, even if this is transient. This means that studies assessing the effectiveness of probiotics must be able to accurately detect in at least semi-quantitative fashion these probiotics organisms in the GI microbiota.

We have demonstrated that erroneous conclusions as to the presence or absence, or relative proportions of, bifidobacteria are likely if universal primers which do not sufficiently complement the target sequence are used. The primers we have designed are able to detect bifidobacteria at low level abundance and can be used semi-quantitatively without distorting the proportions detected of other genera. This primer set can be successfully used in 16S rRNA pyrosequencing based GI microbiota studies.

## Materials and Methods

### PCR primer design

Primers 357F/926R (357F - CCTACGGGAGGCAGCAG, 926R - CCGTCAATTCMTTTRAGT) were assessed for specificity using the ARB software package [38] and the SILVA 108 SSU Ref 16S rRNA database release [39]. Almost all bifidobacteria (as well as some closely related Actinobacteria) were found to have a one base pair mismatch (C → T) to the 926R primer (CCGTCAATT**C**MTTTRAGT, mismatch in bold).

A new ‘bifidobacteria-optimised’ universal primer (926Rb) was therefore synthesised in which a T/C redundancy was incorporated at the mismatch position: CCGTCAATT**Y**MTTTRAGT (where Y is T or C).

### Standard DNA Mixtures

DNA was extracted from pure cultures of *Bifidobacterium dentium*, *Streptococcus pneumoniae* and *Moraxella catarrhalis* using the MP Bio Fast Soil DNA kit®. An extra bead-beating step (40 seconds, speed 6.0 m/s using the FastPrep® FP120 Instrument, MP Biomedicals) was incorporated in order to ensure efficient lysis.

Total genomic DNA concentration was measured using the Quant-iT, PicoGreen DNA assay (Invitrogen).

Pre-defined mixtures using varying proportions of *Bifidobacterium dentium*, *Streptococcus pneumoniae* and *Moraxella catarrhalis* DNAs were prepared (**Table 1**). All three bacterial strains have 4 copies of the 16S rRNA operon. Consequently gene copy number is dependent only on the number of bacteria present.

### Faecal samples

Faecal samples were collected from five healthy term infants at two time points, 4 weeks and 26 weeks of age. The samples were immediately frozen (−12°C to −20°C) prior to transfer (within one week of sampling) to −80°C prior to evaluation.

Total DNA was extracted as described by Matsuki, et al. [40] except that DNA was re-suspended in 0.1 ml of TE (10 mM Tris-HCl, 1 mM EDTA, pH 8.0).

### Barcoded 16S rRNA PCR and pyrosequencing

The V3–V5 regions of the bacterial 16S rRNA gene were amplified using primers 357F with adaptor B from 454 Life Sciences for pyrosequencing: 5′ CTATCCCCTGTGTGCCTTGGCAGTCTCAGCCTACGGGAGGCAGCAG 3′, and either the standard 926R or the ‘bifidobacteria-optimised’ primer 926Rb (Y in place of C, in bold): 5′ CCATCTCATCCCTGCGTGTCTCCGACTCAGNNNNNNNNNNNNCCGTCAATT**C**MTTTRAGT 3′. In addition the reverse primers included the 454 Life Sciences adaptor A and a unique 12 base-pair error-correcting Golay [41] barcode (denoted by ‘Ns’, see Supporting Information S1). This allows multiplexing of samples in a single run. Primers were obtained from Eurofins MWG Operon (Ebersberg, Germany) and HPSF purified.

PCR was carried out in quadruplicate to reduce random mispriming bias [17], and no-template PCR controls were included. Each 25 µl reaction contained 1 µL each of forward and reverse primers (10 µM), 1 µl of template DNA, 0.25 µl of 5 U/µl FastStart HiFi Polymerase (Roche, Mannheim, Germany), 1 µl of 20 g/mL BSA (Sigma, Dorset, United Kingdom), and 6.5 µl of 5 M Betaine (Sigma). PCR reactions were assembled within a PCR hood under clean conditions. Thermal cycling consisted of initial denaturation at 94°C for 2 minutes followed by 30 cycles of denaturation at 94°C for 20 seconds, annealing at 50°C for 30 seconds, and extension at 72°C for 5 minutes. The replicate amplicons were pooled, PEG precipitated [42] (20%, MW 8 000 g/mol) and visualized by staining with ethidium bromide (10 mg/mL) on a 1.0% agarose gel.

### Amplicon quantitation, pooling and pyrosequencing

Amplicons were combined in a single tube in equimolar concentrations. The pooled amplicon mixture was purified twice (AMPure XP kit, Agencourt, Takeley, United Kingdom) and the cleaned pool requantified using the PicoGreen assay. This pool was then diluted in TE such that it contained 10^5^ molecules/µl. 30 µl of this pool was added to the emulsion PCR reaction to attain a ratio of 0.3 molecules of amplicon per bead. Pyrosequencing was carried out on a 454 Life Sciences GS Junior instrument (Roche) following the Roche Amplicon Lib-L protocol.

### Bioinformatics

Shotgun processed data was denoised using AmpliconNoise [43] as part of the QIIME [44] (Quantitative Insights Into Microbial Ecology) package followed by chimera-removal with Perseus [43]. The sequences were aligned using the Greengenes core alignment set as reference (DeSantis, et al 2006) and clustered at 97% sequence identity into OTUs. Representative sequences (most abundant) for each OTU were selected and classified using the Ribosomal Database Project Classifier. Rarefaction was performed so that the number of reads per sample would be identical. Beta diversity assessment of the reads obtained from the faecal samples using the two primer sets was carried out using the weighted UniFrac metric to generate principal coordinate analyses. Identification of OTUs that were significantly different in abundance was carried out in QIIME using a paired T-test with Bonferroni correction.

### Fluorescence *in situ* hybridisation

To enumerate the *Bifidobacterium* genus by means of FISH the 16S rRNA-targeted oligonucleotide probe: Bif164-mod 5′- CATCCGGYATTACCACCC-3′ was used [45], [46]. The probe was commercially synthesized and 5′-labelled with Cy3 (Biolegio B.V., Nijmegen, the Netherlands).

The FISH analysis was performed according to the method of Thiel [47], with some modifications. Briefly, portions of each faecal sample were fixed with 3% paraformaldehyde at 4°C for 16 hours. Following fixation, 1 ml of the cell suspension was centrifuged at 8 000×g for 3 min and the cell pellet resuspended in 500 µl of PBS buffer, mixed with 500 µl of ethanol and then stored at −20°C until use. 3 µl of the fixed-cell suspension of the appropriate dilution (80, 160, 320 and 640 fold dilutions) was applied to chrome gelatine coated 18-well slides (Cel-Line HTC Super cured, Thermo Scientific Portsmouth, NH) and the cell smears were dehydrated for 3 min each in 60%, 80% and 96% ethanol. After hybridization of the probe at 50°C for 16 hours, the slides were washed, dried, counterstained with 4′,6-diamidino-2-phenylindole (DAPI) and mounted with Citifluor AF1 (Citifluor Ltd, London, United Kingdom).

Image acquisition and image analysis was performed using the scan∧R screening station (Olympus, Hamburg, Germany). The count and percentage of labelled bacteria per sample was determined in 25 positions divided over the well by counting all DAPI-stained bacteria and all doubly stained bacteria (DAPI and Cy3) in the same field of view using a quadruple band filter set (Set 84000, Chroma Technology Corp., Brattleboro, VT, USA).

### Data Availability

MIMARKS compliant [39] 16S rRNA amplicon data for the faecal samples has been deposited at MG-RAST [48] under accession numbers 4483884.3 to 4483903.3 (static link http://metagenomics.anl.gov/linkin.cgi?project=329).

### Ethics Statement

The following ethics committees approved all protocols and procedures: National Research Ethics Service Committee, U.K. (Southampton and South West Hampshire) (ref: 05/Q1702/119), SingHealth Centralised Institutional Review Board, Singapore (ref: EC200610143), Gold Coast Health Service District Human Research Ethics Committee, Australia (ref: 200693), and Children, Youth and Women's Health Service Human Research Ethics Committee, Australia (ref: REC 1965/06/10). Parents gave their full written informed consent for faecal sample collection.

## Supporting Information

Supporting Information S1 **Barcoded primer sequences for 16S rRNA PCR.**
(XLSX)

## References

[1] Turnbaugh PJ, Ley RE, Mahowald MA, Magrini V, Mardis ER (2006). An obesity-associated gut microbiome with increased capacity for energy harvest.. Nature.

[2] Forno E, Onderdonk AB, McCracken J, Litonjua AA, Laskey D (2008). Diversity of the gut microbiota and eczema in early life.. Clin Mol Allergy.

[3] Hong PY, Lee BW, Aw M, Shek LP, Yap GC (2010). Comparative analysis of fecal microbiota in infants with and without eczema.. PLoS One.

[4] Zhu Y, Michelle LT, Jobin C, Young HA (2011). Gut microbiota and probiotics in colon tumorigenesis.. Cancer Lett.

[5] Mshvildadze M, Neu J, Shuster J, Theriaque D, Li N (2010). Intestinal microbial ecology in premature infants assessed with non-culture-based techniques.. J Pediatr.

[6] Ivanov II, Littman DR (2011). Modulation of immune homeostasis by commensal bacteria.. Curr Opin Microbiol.

[7] Fujimura KE, Slusher NA, Cabana MD, Lynch SV (2010). Role of the gut microbiota in defining human health.. Expert Rev Anti Infect Ther.

[8] Weisburg WG, Barns SM, Pelletier DA, Lane DJ (1991). 16S ribosomal DNA amplification for phylogenetic study.. J Bacteriol.

[9] Lane DJ, Pace B, Olsen GJ, Stahl DA, Sogin ML (1985). Rapid determination of 16S ribosomal RNA sequences for phylogenetic analyses.. Proc Natl Acad Sci U S A.

[10] Baker GC, Smith JJ, Cowan DA (2003). Review and re-analysis of domain-specific 16S primers.. J Microbiol Methods.

[11] Wang Y, Qian PY (2009). Conservative fragments in bacterial 16S rRNA genes and primer design for 16S ribosomal DNA amplicons in metagenomic studies.. PLoS One.

[12] Cole JR, Wang Q, Cardenas E, Fish J, Chai B (2009). The Ribosomal Database Project: improved alignments and new tools for rRNA analysis.. Nucleic Acids Res.

[13] Sogin ML, Morrison HG, Huber JA, Mark WD, Huse SM (2006). Microbial diversity in the deep sea and the underexplored “rare biosphere”.. Proc Natl Acad Sci U S A.

[14] Meyer AF, Lipson DA, Martin AP, Schadt CW, Schmidt SK (2004). Molecular and metabolic characterization of cold-tolerant alpine soil Pseudomonas sensu stricto.. Appl Environ Microbiol.

[15] Hamady M, Knight R (2009). Microbial community profiling for human microbiome projects: Tools, techniques, and challenges.. Genome Res.

[16] Frank JA, Reich CI, Sharma S, Weisbaum JS, Wilson BA (2008). Critical evaluation of two primers commonly used for amplification of bacterial 16S rRNA genes.. Appl Environ Microbiol.

[17] Polz MF, Cavanaugh CM (1998). Bias in template-to-product ratios in multitemplate PCR.. Appl Environ Microbiol.

[18] Roger LC, McCartney AL (2010). Longitudinal investigation of the faecal microbiota of healthy full-term infants using fluorescence in situ hybridization and denaturing gradient gel electrophoresis.. Microbiology.

[19] Favier CF, de Vos WM, Akkermans AD (2003). Development of bacterial and bifidobacterial communities in feces of newborn babies.. Anaerobe.

[20] Boehm G, Stahl B, Jelinek J, Knol J, Miniello V (2005). Prebiotic carbohydrates in human milk and formulas.. Acta Paediatr Suppl.

[21] Knol J, Scholtens P, Kafka C, Steenbakkers J, Gro S (2005). Colon microflora in infants fed formula with galacto- and fructo-oligosaccharides: more like breast-fed infants.. J Pediatr Gastroenterol Nutr.

[22] Boesten R, Schuren F, Ben AK, Haarman M, Knol J (2011). Bifidobacterium population analysis in the infant gut by direct mapping of genomic hybridization patterns: potential for monitoring temporal development and effects of dietary regimens.. Microb Biotechnol.

[23] van Limpt C, Crienen A (2004). Effect of Colonic Short Chain Fatty Acids, Lactate and Ph on the Growth of Common Gut Pathogens.. Pediatric Research.

[24] Picard C, Fioramonti J, Francois A, Robinson T, Neant F (2005). Review article: bifidobacteria as probiotic agents – physiological effects and clinical benefits.. Aliment Pharmacol Ther.

[25] Ouwehand AC (2007). Antiallergic effects of probiotics.. J Nutr.

[26] van der Aa LB, van Aalderen WM, Heymans HS, Henk Sillevis SJ, Nauta AJ (2011). Synbiotics prevent asthma-like symptoms in infants with atopic dermatitis.. Allergy.

[27] Palmer C, Bik EM, DiGiulio DB, Relman DA, Brown PO (2007). Development of the human infant intestinal microbiota.. PLoS Biol.

[28] Muyzer G, de Waal EC, Uitterlinden AG (1993). Profiling of complex microbial populations by denaturing gradient gel electrophoresis analysis of polymerase chain reaction-amplified genes coding for 16S rRNA.. Appl Environ Microbiol.

[29] Muyzer G, Teske A, Wirsen CO, Jannasch HW (1995). Phylogenetic relationships of Thiomicrospira species and their identification in deep-sea hydrothermal vent samples by denaturing gradient gel electrophoresis of 16S rDNA fragments.. Arch Microbiol.

[30] Peterson J, Garges S, Giovanni M, McInnes P, Wang L (2009). The NIH Human Microbiome Project.. Genome Res.

[31] NIH Human Microbiome Project website.. http://www.hmpdacc.org/doc/HMP_MDG_454_16S_Protocol_V4_2_102109.pdf.

[32] Bland JM, Altman DG (1986). Statistical methods for assessing agreement between two methods of clinical measurement.. Lancet.

[33] Lozupone C, Hamady M, Knight R (2006). UniFrac–an online tool for comparing microbial community diversity in a phylogenetic context.. BMC Bioinformatics.

[34] de Boer R, Peters R, Gierveld S, Schuurman T, Kooistra-Smid M (2010). Improved detection of microbial DNA after bead-beating before DNA isolation.. J Microbiol Methods.

[35] Metchnikoff E (1907). Lactic acid as inhibiting intestinal putrefaction.. The prolongation of life: Optimistic studies.

[36] Macpherson AJ, Harris NL (2004). Interactions between commensal intestinal bacteria and the immune system.. Nat Rev Immunol.

[37] Gilliland S, Morelli L, Reid G (2001).

[38] Ludwig W, Strunk O, Westram R, Richter L, Meier H (2004). ARB: a software environment for sequence data.. Nucleic Acids Res.

[39] Field D, Garrity G, Gray T, Morrison N, Selengut J (2008). The minimum information about a genome sequence (MIGS) specification.. Nat Biotechnol.

[40] Matsuki T, Watanabe K, Fujimoto J, Kado Y, Takada T (2004). Quantitative PCR with 16S rRNA-gene-targeted species-specific primers for analysis of human intestinal bifidobacteria.. Appl Environ Microbiol.

[41] Fierer N, Hamady M, Lauber CL, Knight R (2008). The influence of sex, handedness, and washing on the diversity of hand surface bacteria.. Proc Natl Acad Sci U S A.

[42] Sambrook J, Russell D (2001). Molecular Cloning: A Laboratory Manual.

[43] Quince C, Lanzen A, Davenport RJ, Turnbaugh PJ (2011). Removing noise from pyrosequenced amplicons.. BMC Bioinformatics.

[44] Caporaso JG, Kuczynski J, Stombaugh J, Bittinger K, Bushman FD (2010). QIIME allows analysis of high-throughput community sequencing data.. Nat Methods.

[45] Langendijk PS, Schut F, Jansen GJ, Raangs GC, Kamphuis GR (1995). Quantitative fluorescence in situ hybridization of Bifidobacterium spp. with genus-specific 16S rRNA-targeted probes and its application in fecal samples.. Appl Environ Microbiol.

[46] Satokari RM, Vaughan EE, Akkermans AD, Saarela M, de Vos WM (2001). Polymerase chain reaction and denaturing gradient gel electrophoresis monitoring of fecal bifidobacterium populations in a prebiotic and probiotic feeding trial.. Syst Appl Microbiol.

[47] Thiel R, Blaut M (2005). An improved method for the automated enumeration of fluorescently labelled bacteria in human faeces.. J Microbiol Methods.

[48] Meyer F, Paarmann D, D'Souza M, Olson R, Glass EM (2008). The metagenomics RAST server - a public resource for the automatic phylogenetic and functional analysis of metagenomes.. BMC Bioinformatics.

